# A Rare Moment of Cross-Partisan Consensus: Elite and Public Response to the COVID-19 Pandemic in Canada

**DOI:** 10.1017/S0008423920000311

**Published:** 2020-04-16

**Authors:** Eric Merkley, Aengus Bridgman, Peter John Loewen, Taylor Owen, Derek Ruths, Oleg Zhilin

**Affiliations:** 1 Munk School of Global Affairs and Public Policy, University of Toronto, 1 Devonshire Place, Toronto, ON, M5S 3K7; 2 Department of Political Science, McGill University, 855 Sherbrooke St. West, Room 414, Montreal, QC, H3A 2T7; 3 Max Bell School of Public Policy, McGill University, 680 Sherbrooke St. West, 6^th^ Floor, Montreal, QC, H3A 2M7; 4 School of Computer Science, McGill University, 3480 University St., Room 318, Montreal, QC, H3A 0E9

## Abstract

The COVID-19 pandemic requires an effort to coordinate the actions of government and society in a way unmatched in recent history. Individual citizens need to voluntarily sacrifice economic and social activity for an indefinite period of time to protect others. At the same time, we know that public opinion tends to become polarized on highly salient issues, except when political elites are in consensus (Berinsky, 2009; Zaller, 1992). Avoiding elite and public polarization is thus essential for an effective societal response to the pandemic. In the United States, there appears to be elite and public polarization on the severity of the pandemic (Gadarian et al., 2020). Other evidence suggests that polarization is undermining compliance with social distancing (Cornelson and Miloucheva, 2020). Using a multimethod approach, we show that Canadian political elites and the public are in a unique period of cross-partisan consensus on important questions related to the COVID-19 pandemic, such as its seriousness and the necessity of social distancing.

The COVID-19 pandemic requires an effort to coordinate the actions of government and society in a way unmatched in recent history. Individual citizens need to voluntarily sacrifice economic and social activity for an indefinite period of time to protect others. At the same time, we know that public opinion tends to become polarized on highly salient issues, except when political elites are in consensus (Berinsky, 2009; Zaller, 1992). Avoiding elite and public polarization is thus essential for an effective societal response to the pandemic. In the United States, there appears to be elite and public polarization on the severity of the pandemic (Gadarian et al., 2020). Other evidence suggests that polarization is undermining compliance with social distancing (Cornelson and Miloucheva, 2020). Using a multimethod approach, we show that Canadian political elites and the public are in a unique period of cross-partisan consensus on important questions related to the COVID-19 pandemic, such as its seriousness and the necessity of social distancing.

## Elite Cues and Public Opinion

The theory that political elites have the power to shape public attitudes has a long history in political science. Citizens “follow the leader” in part as a low-information shortcut to form opinions likely to be in line with their interests (Mondak, 1993) or to reaffirm their deeply rooted partisan identities (Bakker et al., 2019). Observational and experimental research has thus found public attitudes to be highly responsive to cues from parties (Berinsky, 2009; Lenz, 2012; Mondak, 1993), especially on novel, “hard” issues where citizens are dependent on the news media for information (Tesler, 2018; Zaller, 1992).

Polarization is often the norm on highly salient political issues, and this has been true for matters of science as well. For example, there is substantial evidence that divided political elites polarized American attitudes toward climate science (Carmichael and Brulle, 2017; Merkley and Stecula, forthcoming; Tesler, 2018). Polarization can only be avoided if elites send signals of consensus (Berinsky, 2009; Zaller, 1992). Most research on cue-taking has been situated in the United States, but some work has illustrated the importance of elite cues comparatively (Bischof and Wagner, 2019) and in Canada specifically (Merolla et al., 2016).

The implications of elite disagreement on an issue like the COVID-19 pandemic are considerable. Divided parties send polarizing signals to the mass public that could undermine efforts to fight the virus. In the United States, Republican officials voiced skepticism about the severity of the pandemic early in the crisis, and attitudes toward COVID-19 are heavily polarized, perhaps as a result (Gadarian et al., 2020). The past few decades have seen polarization increase in Canada (Cochrane, 2015). Here, we evaluate the degree to which the politics in Canada regarding COVID-19 can be characterized by partisan polarization.

## Data and Methods

We employ data from the social media accounts of federal Members of Parliament (MPs), Google search trends, and public opinion surveys to evaluate the response to COVID-19 by Canadian political elites and the mass public. To assess elite cues, we collected all tweets from MPs who use Twitter (292 accounts, with a total of 33,142 tweets since January 1, 2020). We used keyword searches to classify tweets into one or more topics. We calculate the share of MP tweets mentioning COVID-19 by party and then benchmark these series against other issues (environment and immigration). Hand-coding was done on all tweets related to COVID-19 in order to identify signals downplaying the severity of the crisis and messages promoting social distancing.

Following the elite cue analysis, we gauge the relationship between partisanship and concern about COVID-19 at both the aggregate and individual levels. We collected Google search trends for the search term “coronavirus” in the first half (1–14) and second half (15–31) of March. We average these two periods together at the municipal level (*N* = 87). These data show the *relative* difference in search interest in the coronavirus between municipalities. We obtained municipal-level estimates of the Conservative party's (CPC) vote share in 2015, as well as population size (logged), population density (logged), average median income, and share of population with a postsecondary education.^1^ We construct an urban index, with population size and density, and a socio-economic status (SES) index, from education and income levels (both scaled 0–1). We estimate a model predicting relative search interest in the coronavirus with Conservative party vote share, the urban and SES indices, and provincial fixed effects with robust standard errors.

We conducted a survey of 2,499 Canadian citizens 18 years and older from the online sample provider Dynata fielded from April 2 to April 6. National level quotas were set on region (that is, Atlantic, Quebec, Ontario, West), age, gender and language. Data were weighted within each region of Canada by gender and age, based on data from the 2016 Canadian census. We asked our respondents about their level of concern with COVID-19 and how serious of a threat they believed it to be for themselves and for Canadians in general. We create a COVID-19 severity index from these responses, scaled from 0 to 1.

We also asked our respondents whether or not they have engaged in a series of social distancing behaviours. We use principal components analysis to identify two dimensions that run through these responses, roughly corresponding to their offline (for example, avoiding large crowds) and online (for example, working from home) social distancing. Consequently, we construct two indices of social distancing from these factors, scaled from 0 to 1. Factor loadings can be found in Table A1 of the appendix. We estimate models regressing our severity and social distancing indices on partisanship and left–right ideology,^2^ with controls for income, education, age, religiosity, urban residence, gender, French language and region.

## Results

**Figure 1.**
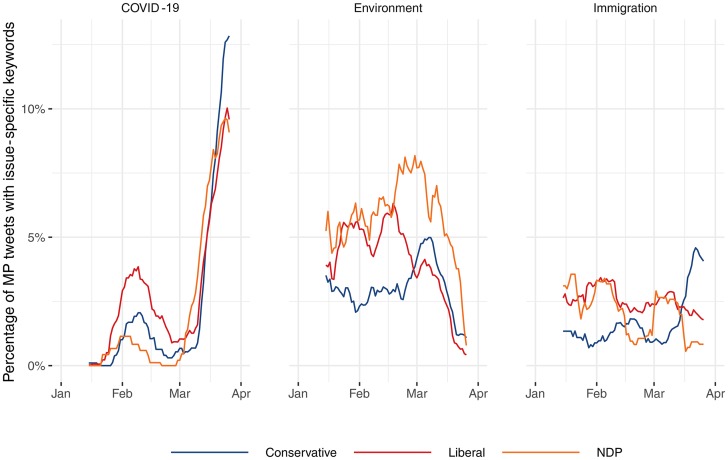
Rolling Percentage (*n* = 15) of Tweets Focused on COVID-19, the Environment and Immigration from Federal Members of Parliament as Identified by Keyword Searches

Quantitative and qualitative reviews of MP tweets from January 1 to March 28 from the three national parties with official party status indicate that political elites in Canada have presented a united front on the nature and severity of the COVID-19 pandemic. Figure 1 shows there was relatively little focus on the virus until early March, at which point discussion from all parties exploded, with tweets on other issues that have historically been important in the Canadian context seeing a sizable reduction. More information on the keywords for the automated analysis and tweet frequency by party can be found in the online supplement.

Counts do not tell the whole story, however. It could be that increased attention to the issue was principally partisan in nature. We thus qualitatively coded all tweets mentioning COVID-19 in our sample (*N* = 1,260). Our coding indicates that members from all three parties heavily emphasized (in roughly equal proportions) the importance of social distancing measures and proper hygiene practices, like handwashing and not touching one's face. Additionally, there were no tweets from MPs of any party that indicated concerns about COVID-19 were overblown or exaggerated, nor were there any that spread misinformation about COVID-19 (for example: vitamins and high temperatures as cures, the human consumption of bats as a cause, and COVID-19 being no more dangerous than the flu). Coding criteria can be found in the online supplement.

### Aggregate-Level Analyses

**Figure 2.**
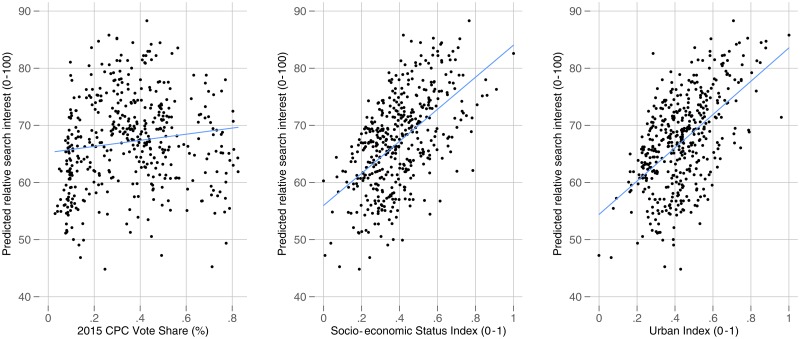
Predicted Municipal-Level Search Interest in Coronavirus over Conservative Party Vote Share (left), Socio-economic Status Index (centre) and Urban Index (right)

This elite consensus on the seriousness of COVID-19 is reflected in aggregate-level partisan differences in search traffic on the coronavirus. The estimates from our model are provided in Table A2 in the appendix. We plot our model predictions for Canadian municipalities in Figure 2 across Conservative party vote share (left panel) and our socio-economic (centre panel) and urban status indices (right panel). There is no significant association between Conservative party vote share and search interest in the coronavirus. Interest in the coronavirus among municipalities is much more strongly determined by socio-economic (*p* < 0.001) and urban characteristics (*p* < 0.001).

### Individual-Level Analyses

**Figure 3.**
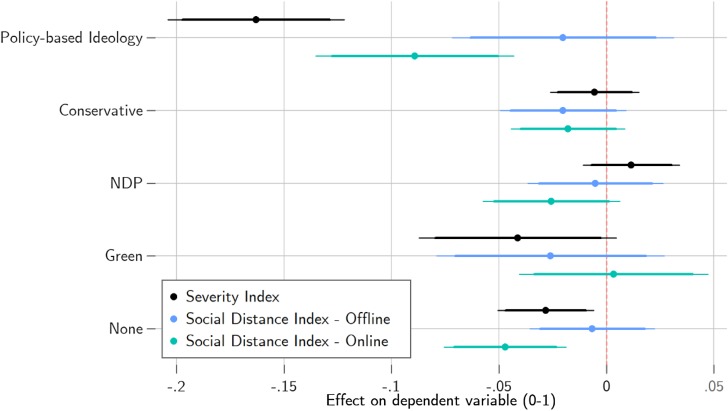
Effects of Ideology and Partisanship on Severity and Social Distance Indices

*Note:* 95 and 90 per cent confidence intervals. Liberal partisans are reference category for partisanship. Controls for income, education, age, religiosity, gender, language and region.

The null result highlighted by our aggregate-level analyses are also reflected in individual-level COVID-19 risk perceptions and self-reported social distancing practices. Partisans of the Liberals, Conservatives and the New Democratic Party (NDP) are not significantly different in their social distancing practices or in their perceptions of COVID-19 severity, after accounting for ideology and demographics, though nonpartisans do generally score lower. Our model estimates for partisanship and ideology are plotted in Figure 3, and the full model estimates can be found in Table A2.

Ideology does appear to matter. Crossing the full range of this index is expected to reduce one's perceptions of COVID-19 severity by 0.2 points (*p* < 0.01). Someone who is consistently left-wing is expected to score 0.87 on the 0–1 scale, compared to 0.71 for those who are consistently right-wing in their beliefs. Likewise, online social distancing is expected to decrease from 0.41 to 0.31 (*p* < 0.01), on the 0–1 scale, though there appears to be no effect on offline social distancing. Ideology, rather than partisan identity or elite cue-taking, appears to be driving small partisan differences in COVID-19 attitudes and social distancing practices.

## Discussion

The above findings suggest that both Canadian elites and the mass public are in a moment of cross-partisan consensus on COVID-19. MPs of all parties have increasingly emphasized the crisis and reinforced the messages of mainstream expert communities. At the aggregate level, there is no evidence of a relationship between the partisan leanings of municipalities and interest in the coronavirus; at the individual-level, very small partisan differences generally disappear when controlling for ideology and demographics. Unlike in the United States, response to the coronavirus is not structured by partisanship, at least at the moment. As the crisis wears on and economic costs mount, it is essential that Canadian parties maintain a united front to avoid eroding this unique moment of societal consensus.

## Notes

1 The vote share estimates use a real weighted interpolation to map 2015 polling-station-level results into census subdivision (that is, municipal) boundaries. Municipal-level data were generously shared by Jack Lucas. Other municipal-level measures are publicly available at https://dataverse.scholarsportal.info/dataset.xhtml?persistentId=doi:10.5683/SP2/947REI.

2 Measured as an index of five policy questions coded in a left–right direction, scaled from 0 to 1. Descriptive statistics and variable descriptions for aggregate and individual analyses can be found in Table S1 of the online supplement.

## Supplementary material

For supplementary material accompanying this paper visit http://dx.doi.org/10.1017/S0008423920000311. click here to view supplementary material

## Appendix

**Table A1.** Factor loadings

Variable	Factor 1	Factor 2	Uniqueness
Worked from home	−0.164	0.733	0.437
Avoided bars, restaurants and crowds	0.767	−0.001	0.412
Avoided grocery stores at peak times	0.601	0.210	0.595
Avoided in-person contact	0.707	0.068	0.495
Stocked up on provisions	0.258	0.404	0.770
Kept distance of two metres	0.767	−0.001	0.412
Switched to virtual meetings	0.266	0.662	0.492
Switched to online shopping	0.238	0.614	0.566
Avoided domestic travel	0.715	0.158	0.465
Avoided public transit	0.705	0.187	0.468

*Note:* We generate two scales of social distancing: offline and online. Offline consists of avoiding bars, restaurants and crowds; avoiding grocery stores at peak times; avoiding in-person contact; keeping a distance of two metres; avoiding domestic travel; and avoiding public transit. Online consists of working from home; switching to virtual meetings; and switching to online shopping.

**Table A2.** Regression Estimates, OLS

	Aggregate		Individual-Level					
			*Severity*		*Offline SD*		*Online SD*	
	Coef.	SE	Coef.	SE	Coef.	SE	Coef.	SE
CPC vote share	−5.88	8.24						
SES	29.32***	5.35						
Urban	22.72***	7.19						
Ideology			−0.02***	0.00	0.00	0.00	−0.01***	0.00
Conservative PID			−0.01	0.01	−0.02	0.01	−0.02	0.01
NDP PID			0.01	0.01	−0.01	0.02	−0.03	0.02
Green PID			−0.04*	0.02	−0.03	0.03	0.00	0.02
Other PID			−0.02	0.02	0.03	0.03	−0.01	0.03
No PID			−0.03**	0.01	−0.01	0.01	−0.05***	0.01
Income			0.01**	0.00	−0.01***	0.00	0.02***	0.00
Education			−0.00*	0.00	0.02***	0.00	0.02***	0.00
Age			0.00***	0.00	0.01***	0.00	−0.00***	0.00
Religiosity			0.02***	0.00	−0.02***	0.00	0.01	0.00
Urban			0.00	0.00	−0.01***	0.00	0.01*	0.00
Female			0.00	0.01	0.10***	0.01	0.02**	0.01
French			−0.08***	0.02	−0.09***	0.03	0.05*	0.03
Constant	31.02***		0.84***	0.03	0.49***	0.05	0.23***	0.04
Fixed effects	Province		Region		Region		Region	
*R*^2^	0.60		0.12		0.18		0.15	
*N*	87		2250		2250		2250	

*Note:* SD = social distancing; robust standard errors for aggregate-level model. PID = partisan identification.

* *p* < 0.1; ** *p* < 0.05; *** *p* < 0.01.
