# Supplementary material for: A Rare Moment of Cross-Partisan Consensus: Elite and Public Response to the COVID-19 Pandemic in Canada
Source: Can J Polit Sci. 2020 Apr 16:1–8. doi: 10.1017/S0008423920000311 (PMC7235300; doi:10.1017/S0008423920000311)
Supplement: Supplementary file 1 [file S0008423920000311sup001.docx]

**Supplementary Materials**

*A Rare Moment of Cross-Partisan Consensus:
Elite and Public Response to the COVID-19 Pandemic in Canada*

An elite consensus requires that elected members across parties are sharing similar messaging and that it is not just a small group of Members of Parliament who are tweeting. We show below two additional plots which indicate that MPs do tweet at differing levels, but that there few outliers. Figure S1 shows the density curve of number of tweets. Conservative MPs tend to tweet less than NDP and Liberal ones and there are a few more-frequent tweets but generally tracked MPs tweeted between 100-200 times during the period under examination. Figure S2 shows that MPs from different parties generally tweet at similar rates.

**Figure S1.** Density plot of volume of tweets from Conservative, Liberal, and New Democrat Members of Parliament.

**Figure S2.** Frequency plot of average daily volume of tweets from Conservative, Liberal, and New Democrat Members of Parliament.

The following dictionary was used to detect thematic English-language tweets. A keyword search was effected over all tweets by Members of Parliament from the Liberal, Conservative, and New Democratic Parties.

| Theme | Associated words |
| --- | --- |
| COVID-19 | 'covid', 'virus', 'social distanc', 'quarantine', 'ventilator', 'n95', 'n-95', 'pandemic', 'epidemic', 'corona', 'community spread', 'self-isolate', 'self isolate', 'herd immunity', 'flatten the curve' |
| Immigration | 'migration', 'refugee', 'asylum', 'border', 'intolerance', 'discrimination', 'cultural', 'diversity', 'irregular', 'integrat', 'settle', 'deportation', 'illegal crossing' |
| Environment | 'environment','climat','green', 'clean', 'carbon', 'smog', 'pollution', 'global warming', 'paris accord' |

All tweets that were identified as having one or more of the keywords associated with COVID-19 were then manually annotated by a single coder for the following features:

| Feature | Description |
| --- | --- |
| Downplaying | Any content that downplays the severity of the crisis or suggests that social distancing measures are not effective for limiting the spread of the virus. |
| Misinformation | Any content that shares medical misinformation. As there is much uncertainty surrounding the virus, here the following misinformation was checked for: consumption of bats started pandemic in China, the virus will end in the summer in a manner similar to the seasonal flu, homeopathic cure for the virus, vitamin C as a cure for the virus, that the virus is no worse than the seasonal flu, that high temperatures produced by things like hair dryers can easily kill the virus, that big pharma is withholding a vaccine, or that COVID-19 is a Chinese bioweapon. |
| Social distance | Any content that promotes social distancing measures including maintaining at least 2m, coughing into your arm, avoiding large social gatherings, washing your hands, and not touching your face. |

**Table S1.** Descriptive Statistics

| **Aggregate Data** | **Description** | **Mean** | **SD** | **Min** | **Max** |
| --- | --- | --- | --- | --- | --- |
| 2015 Conservative Party vote share |  | 0.32 | 0.14 | 0.05 | 0.70 |
| Urban index | 0-1 | 0.44 | 0.15 | 0 | 1 |
| Population density | Logged | 6.60 | 1.10 | 2.83 | 8.61 |
| Population | Logged | 11.81 | 0.97 | 9.15 | 14.82 |
| SES index | 0-1 | 0.40 | 0.16 | 0 | 1 |
| Median household income |  | 76120 | 17027 | 50227 | 119905 |
| % postsecondary |  | 65.33 | 6.68 | 52.38 | 80.62 |
| **Survey Data** | **Description** | **Mean** | **SD** | **Min** | **Max** |
| Severity index | 0-1 | 2.33 | 0.62 | 0 | 1 |
| COVID-19 concern | Not at all (0); A little (1); Somewhat (2); Very (3) | 2.53 | 0.71 | 0 | 3 |
| COVID-19 serious - Self | Not at all (0); Not very (1); Somewhat (2); Very (3) | 2.10 | 0.80 | 0 | 3 |
| COVID-19 serious - Canadians | Not at all (0); Not very (1); Somewhat (2); Very (3) | 2.56 | 0.61 | 0 | 3 |
| Offline social distancing | 0-1 | 0.75 | 0.26 | 0 | 1 |
| Online social distancing | 0-1 | 0.37 | 0.24 | 0 | 1 |
| Worked from home | =1 | 0.42 | 0.49 | 0 | 1 |
| Avoid bars, restaurants, and crowds | =1 | 0.84 | 0.36 | 0 | 1 |
| Avoid grocery stores at peak times | =1 | 0.74 | 0.44 | 0 | 1 |
| Avoid in-person contact | =1 | 0.80 | 0.40 | 0 | 1 |
| Stock up on provisions | =1 | 0.45 | 0.50 | 0 | 1 |
| Keep 2 metres distance | =1 | 0.84 | 0.36 | 0 | 1 |
| Switch to virtual meetings | =1 | 0.38 | 0.49 | 0 | 1 |
| Switch to online shopping | =1 | 0.33 | 0.47 | 0 | 1 |
| Avoid domestic travel | =1 | 0.71 | 0.45 | 0 | 1 |
| Avoid public transit | =1 | 0.67 | 0.47 | 0 | 1 |
| Liberal PID | =1 | 0.32 | 0.47 | 0 | 1 |
| Conservative PID | =1 | 0.26 | 0.44 | 0 | 1 |
| NDP PID | =1 | 0.12 | 0.33 | 0 | 1 |
| Green PID | =1 | 0.05 | 0.22 | 0 | 1 |
| Other PID | =1 | 0.04 | 0.20 | 0 | 1 |
| None PID | =1 | 0.20 | 0.40 | 0 | 1 |
| Left-right ideology | 0-1 | 0.42 | 0.22 | 0 | 10 |
| Religiosity | Not at all important (0); Not very important (1); Somewhat important (2); Very important (3) | 1.35 | 1.12 | 0 | 3 |
| Education | No schooling (0); Some elementary (1); Completed elementary (2); Some secondary (3); Completed secondary (4); Some technical (5); Completed technical (6); Some university (7); Bachelors (8); Masters (9); Professional or doctorate (10) | 6.26 | 2.04 | 0 | 10 |
| **Survey Data** | **Description** | **Mean** | **SD** | **Min** | **Max** |
| Income | No income (0); $1-30,000 (1); $30,001-60,000 (2); $60,001-90,000 (3); $90,001-110,000 (4); $110,001-150,000 (5); $150,001-200,000 (6); More than $200,000 (7) | 3.10 | 1.65 | 0 | 7 |
| Age | In years | 47.65 | 16.52 | 18 | 88 |
| French Language | =1 | 0.20 | 0.40 | 0 | 1 |
| Female | =1 |  |  |  |  |
| Urban/rural |  |  |  |  |  |
| Atlantic | Newfoundland, PEI, New Brunswick, Nova Scoria | 0.07 | 0.25 | 0 | 1 |
| Quebec | =1 | 0.23 | 0.42 | 0 | 1 |
| Ontario | =1 | 0.38 | 0.49 | 0 | 1 |
| West | British Columbia, Alberta, Saskatchewan, and Manitoba | 0.32 | 0.47 | 0 | 1 |
